# Simultaneous imaging of neural activity in three dimensions

**DOI:** 10.3389/fncir.2014.00029

**Published:** 2014-04-03

**Authors:** Sean Quirin, Jesse Jackson, Darcy S. Peterka, Rafael Yuste

**Affiliations:** Department of Biological Sciences, Columbia UniversityNew York, NY, USA

**Keywords:** three-dimensional imaging, calcium imaging, volume imaging, spatial-light-modulator, brain activity map

## Abstract

We introduce a scanless optical method to image neuronal activity in three dimensions simultaneously. Using a spatial light modulator and a custom-designed phase mask, we illuminate and collect light simultaneously from different focal planes and perform calcium imaging of neuronal activity *in vitro* and *in vivo*. This method, combining structured illumination with volume projection imaging, could be used as a technological platform for brain activity mapping.

## Introduction

Optical imaging of neural activity has several advantages over alternative strategies such as patch electrodes or electrode arrays. First, it is minimally invasive and allows for the monitoring of large ensembles of neurons with single-cell resolution (Yuste and Katz, 1991). In addition, it is compatible with a large variety of functional sensors (voltage, calcium or metabolic indicators, either exogenous or genetically encoded), and can be used for chronic imaging of defined cell populations *in vitro* or *in vivo* (Grynkiewicz et al., 1985; Kralj et al., 2012; Chen et al., 2013).

However, some key weaknesses remain for functional imaging of neuronal cell activity. From the very first microscope designed by Leeuwenhoek, image acquisition is typically limited to a single plane, while nearly all biological structures are three-dimensional, thus requiring sequential scanning for volumetric imaging. As a result of this sequential scanning, the sampling rate over the volume is slow relative to neuronal activity (1–100 ms). Moreover, in highly scattering tissue, although two photon excitation has afforded single cell resolution and imaging below superficial layers (Horton et al., 2013), images are still mostly generated by serially scanning a single beam (for exceptions see Gobel et al., 2007; Cheng et al., 2011; Katona et al., 2012). Temporal focusing techniques can deliver high-fidelity illumination patterns into scattering tissue within a limited field-of-view but still require axial scanning with a moving objective, potentially coupling mechanical motion into the sample (Schrödel et al., 2013). Despite recent advances to increase the image acquisition speed, few proposals are scalable toward meeting the ultimate goal of fast, population voltage imaging with single cell resolution in scattering tissue—a task that would require millisecond temporal resolution across wide spatial areas (Alivisatos et al., 2012). Here we present an alternative to traditional scanning-based imaging by taking advantage of programmable three-dimensional illumination with spatial light modulators (SLM) to simultaneously excite neurons located at different focal points, together with a wavefront coded imaging approach that enables us to collect light from all focal points simultaneously. Our technique therefore supersedes scanning by illuminating and collects light from the neurons of interest in parallel.

## Materials and methods

### Wavefront coding for volume projection microscopy

**Figure 1:**
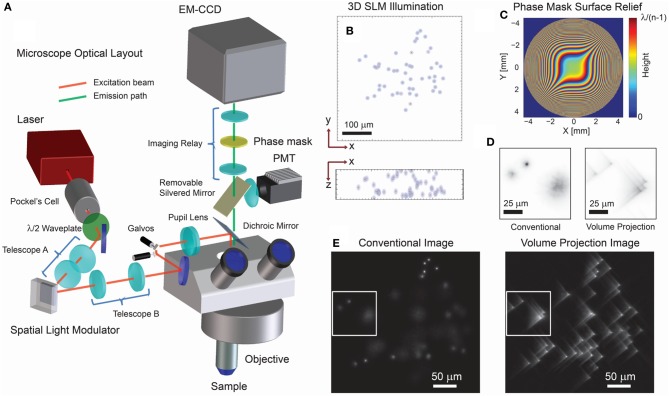
**Optical layout and characteristics of 3D projection-based imaging**. **(A)** The optical configuration comprises an illumination path which incorporates a SLM for 3D structured illumination and a modified imaging path using a phase mask (PM) to suppress the imaging effect of defocus. **(B)** An example 3D illumination pattern using a 20x/0.5NA objective. **(C)** Ideal surface profile modulation of the phase mask. **(D)** Experimental imaging results using a transparent fluorescent slab demonstrate that the volume projection imaging path results in clearer separation of the region-of-interest signals when compared with the conventional imaging path. Note that the contrast conserves the number of photons in each image. **(E)** Wide-field imaging results comparing the conventional and volume projection imaging techniques. Note that each image is normalized to the respective peak signal.

While SLM based multi-site excitation has been coupled to widefield microscopy before, it has been limited to 2D or planar acquisition (Nikolenko et al., 2008; Anselmi et al., 2011). In contrast, we use a phase-only SLM to create multiple well-defined beamlets of light that optically sample many neurons throughout the sample volume. The wavefront coded imaging system then creates a 2D projection of this extended sample volume onto the detector plane. Our wavefront coded imaging system is physically realized by a phase mask placed between an image relay of the microscope pupil (Figure 1A) and is optimized for high-speed, three-dimensional data acquisition in what we call Volume Projection Imaging (VPI) (Dowski and Cathey, 1995; Quirin et al., 2013). Cells are no longer restricted to a single plane and can be freely distributed throughout the three-dimensional sample (Figure 1B). We note that although in some samples (transparent or effectively sparse), single photon excitation is sufficient, we regard two-photon capabilities as essential in turbid environments and for allowing precise modulation of activity when coupled with optogenetics or caged compounds (Papagiakoumou et al., 2010; Packer et al., 2012).

Operationally, in our method, high spatial-resolution structural volume data is first acquired via conventional single beam two-photon raster scanning. This volume data is then processed to identify the cells to be targeted by the SLM. Using these targets, we generate a hologram (phase pattern) that when displayed on the SLM creates the 3D structured illumination which illuminates the regions of interest (ROIs) in the volume (see Quirin et al., 2013 and references therein). Data is acquired by imaging the fluorescent activity of all of the ROIs simultaneously by the cubic phase wavefront coded imaging system regardless of their locations in 3D space (Cathey and Dowski, 2002; Quirin et al., 2013). The deterministic illumination provided by the SLM provides a priori knowledge which can be used to extract additional information from the collected image, as explained below.

For simultaneous 3D signal acquisition, our wavefront coded imaging system increases the standard limited depth-of-field, while maintaining the full aperture, and hence collection efficiency, of the objective. This custom imaging system uses a phase-only optical mask in the imaging path to effectively null defocus effects in the image (Dowski and Cathey, 1995). The effect of this is to greatly reduce the axial dependence of the point spread function (PSF) (i.e., the image is invariant to 3D position), which allows us to record an aberrated line-of-sight projection of the sample volume onto the image plane. This phase mask is described by the complex amplitude profile,

(1) $$p\left(x,y\right)=\{\begin{array}{c} e^{i2\pi \alpha \left(x^{3}+y^{3}\right)}\\ 0 \end{array}\begin{array}{c} \text{ if }x^{2}+y^{2}\leq 1\\ \text{ otherwise} \end{array}\text{​},$$

where we used values of α = 17 and 12 for *M* = 20x/0.5NA and 40x/0.8NA objectives, respectively, and *x*, *y* are the normalized coordinates of the microscope pupil (Figure 1C).

The trade-off for this defocus invariance is a decreased contrast in the focused image (Figure 1D) (Cathey and Dowski, 2002; Quirin et al., 2013). As a result, the acquired wide-field image exhibits a characteristic blur (e.g., an aberrated Point Spread Function) which is now essentially invariant regardless of the three-dimensional location of the ROI (Figure 1E). This blur is nonetheless spatially restricted, and reduction of the out-of-focus PSF size compared with traditional imaging allows for a much higher local spatial density of ROIs (Figure 1D). Now, fluorescence signals from throughout the volume can be simultaneously acquired without loss of contrast due to out-of-focus collection (Figure 1E). As an additional benefit, the data volume is projected onto a 2D image plane thus reducing the total data throughput required during acquisition. In principle, this image can be digitally restored to diffraction-limited resolution by use of deconvolution methods—however, these steps are not necessary here. Instead we report results using a least-squares optimal signal extraction method described in section Targeting Calibration, Image Acquisition and Signal Reconstruction, which had not been applied in earlier methods.

### Microscope setup

**Figure 2:**
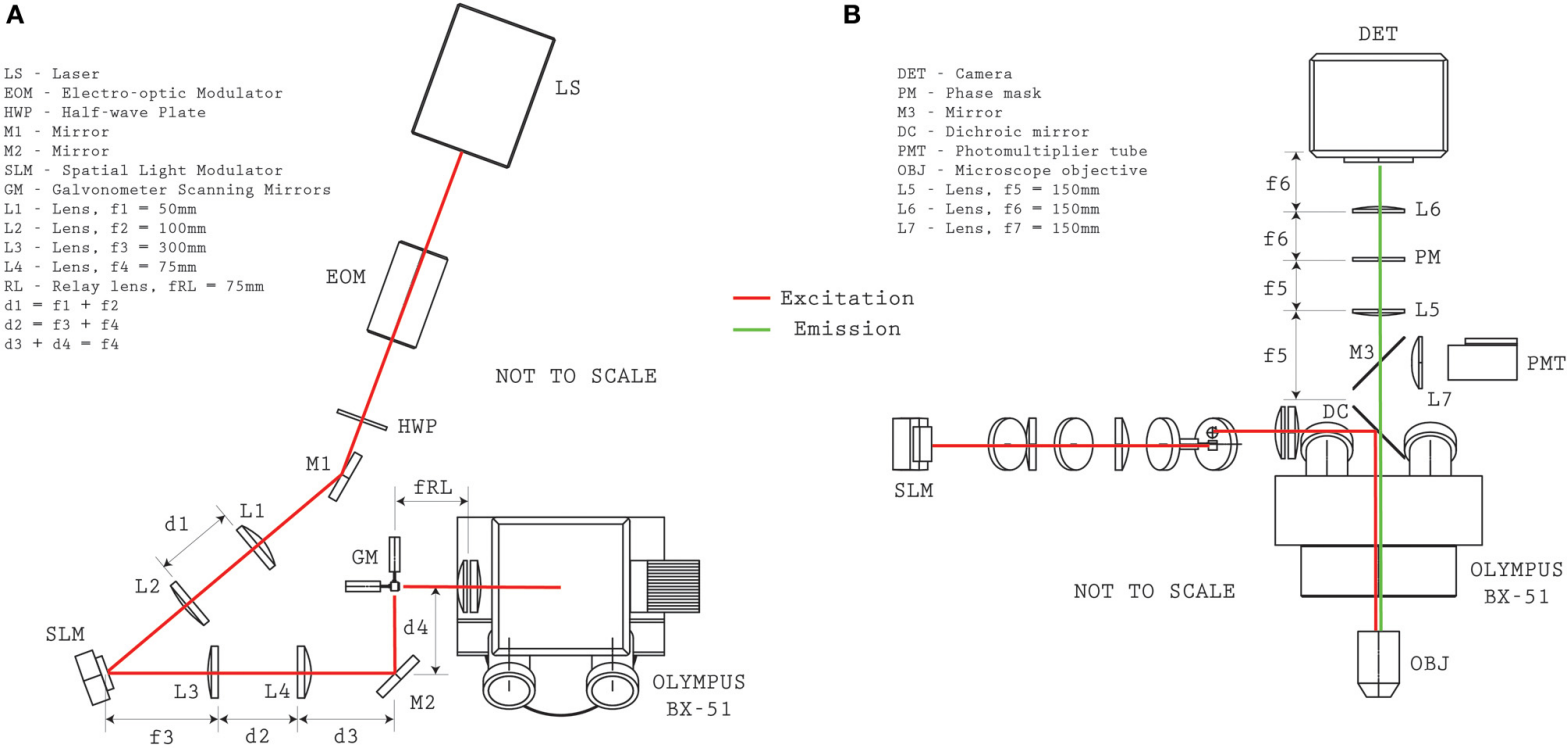
**Optical system configuration**. Plan **(A)** and elevation **(B)** views provide the relative distances between optical elements for reproducing the microscope configuration.

The structured-illumination microscope with VPI uses a single laser for exciting fluorescence of either the calcium indicator dye (Fura2-AM) or a genetically-encoded calcium indicator (GCaMP5G). A detailed optical layout of the system is given in Figure 2. The laser source is a Coherent Mira HP (~140 fs pulses, 80 MHz, linear polarization) which can be manually tuned between ~720 and 1100 nm. At λ = 800 nm, the system provides 3.8 W and at λ = 920 nm, 2.2 W (for Fura2-AM and GCaMP5G excitation, respectively). The output beam was directed through an electro-optic (EO) modulation device (Pockels cell) to modulate intensity on the sample (ConOptics EO350-160). A broadband λ /2 waveplate (Thorlabs AHWP05M-980) was located after the EO modulator to rotate the polarization state to be parallel with the active axis of the Spatial Light Modulator (Boulder Nonlinear Systems, XY-512) located further downstream. A shutter (Vincent Associates, LS6Z2) was placed in the beam path in order to control illumination state condition (i.e., “ON” and “OFF”). A 1:2 telescope (f1 = 50 mm, f2 = 100 mm, Thorlabs plano-convex lenses) scales the optical beam to approximately 8 mm to fill the active area of the SLM. The expanded beam is then redirected to the SLM by a periscope. The SLM has a custom look-up table which was experimentally determined at both wavelengths reported for use. The angle of incidence of the illumination beam to the SLM was ~10°. A 4:1 telescope (f3 = 300 mm, f4 = 75 mm, Thorlabs plano-convex lenses) reduces the image of the SLM onto a set of galvanometer mirrors (Cambridge 6210H, 4 mm open aperture). The galvanometer mirrors are located conjugate to the microscope objective pupil of an Olympus BX-51 microscope by use of an Olympus pupil transfer lens (f5 = 50 mm) and the mounted tube lens (fTL = 180 mm). Two microscope objectives were used for the experiments presented in the manuscript—an Olympus 40x/0.8NA WI (slice experiments) and an Olympus 20x/0.5NA WI objective (*in vivo* experiments), though many other objectives performed similarly during informal testing. In practice, due to transmission losses from the passive and active optical elements, such as the EO modulator and the SLM (from pixel-cross talk, pixel fill factor, etc.), and from beam vignetting at the telescope after the SLM, the overall transmission efficiency from laser to sample was approximately 30%.

When operating in conventional two-photon raster scanning mode, Olympus Fluoview was used to control the galvanometers and digitize the signal from a Photo-Multiplier Tube (PMT—Hamamatsu H7422P-40) located above the microscope. During PMT imaging a removable silvered mirror (M3, mounted in Thorlabs LC6W and LB4C) redirect the collected fluorescence emission into the PMT module.

To operate in volume projection mode, custom software was developed under MATLAB (The MathWorks, Natick, MA) which loads the correct look-up table and generates a hologram for the SLM that targets the selected ROIs located throughout the sample volume. When the illumination shutter is opened, the sample is illuminated with a custom light pattern that addresses the targeted cell somata. For VPI acquisition, the PMT redirection mirror, M3, is removed and the fluorescence emission is passed through an optical relay system with the custom phase mask. The optical relay consists of a 1:1 telescope (f4f = 150 mm, Thorlabs 2” achromatic doublets) with a custom designed surface relief patterned onto a 1” diameter quartz plate that is placed a distance f4f behind the first lens. Note that no amplitude modulation is necessary (i.e., the mask is transparent), critical for high sensitivity imaging, and that the surface relief of the mask is described by,

(2) $$h\left(x,y\right)=\left(\frac{\arg\left\{p\left(x,y\right)\right\}}{2}\pi \right)\left(\frac{\lambda }{n-1}\right)$$

where *h* is the height, *p*(*x*, *y*) is the complex function describing the pupil (Equation 1), λ is the emission wavelength and *n* is the refractive index of the substrate (here, we use fused quartz with *n* = 1.462 at λ = 510 nm). In practice, our realized surface relief is a discrete 8-level approximation of Equation 2 with α = 200 over an 18 mm diameter, with an optimal λ ≈ 510 nm. A 510 ± 30 nm chromatic filter was located immediately after the phase mask. An imaging detector (Andor iXon Ultra 897, electron-multiplying CCD) is located in the imaging plane of the 1:1 relay system.

### Targeting calibration, image acquisition and signal reconstruction

Precise targeting of the ROIs (neurons) was facilitated by a 3D calibration of the SLM projection patterns. A calibration phantom was created using a 2% agarose mixture with a dye solution (yellow highlighter dye) at a 1:1 ratio. Standard projection grids are projected by the SLM at up to 20 z-positions through the volume of interest and the resulting fluorescence image was used to calculate the affine transformation that maps the SLM-based illumination pattern to the EM-CCD. A 3rd-order polynomial fit of each element from the axially dependent affine matrix was used to approximate the axial evolution of the transform and stored for later use (Quirin et al., 2013). A pollen grain slide was similarly used to calibrate the axial dependent, affine transformation from the EM-CCD image to the PMT image frame. The slide was translated through the volume range of interest and an EM-CCD image and PMT image were acquired for comparison. An automated fitting routing estimated the axial-dependent affine transform matrix and, as with the SLM to EM-CCD transform, a 3rd-order polynomial fit is made to each element of the matrix. Matrix multiplication of the PMT to EM-CCD and the EM-CCD to SLM transforms yield the coordinates to load onto the SLM for precise targeting. Though it is known that any errors in matching the refractive index of the phantom material to that of the biological sample will result in uncorrected spherical aberration for the structured illumination pattern and that real tissue has inherent inhomogeneity that will further aberrate the illumination beamlets, in practice we find that both of these factors appear negligible when using the above calibration prescription for the axial ranges and samples described in this paper.

Images acquired on the imaging detector in volume projection mode were processed using custom analysis software written in MATLAB (The Mathworks, Natick, MA). Volume projection acquisition mode consists of generating the hologram illumination pattern for the SLM (see Quirin et al., 2013, and references therein for details), setting the EO modulator for appropriate illumination power, opening the shutter and acquiring time-series images that were saved as 16-bit TIFs (image acquisition under the Andor Solis environment). Because the magnified image of the neurons was relatively large (~200 μm per soma) when using an *M* = 20× objective compared to the imaging detector pixel size (16 μm), pixel binning of 4×4 was routinely used to improve SNR without any appreciable loss of spatial resolution performance. Using the calibration matrices described above, an experimentally measured PSF (taken from the calibration process) is convolved with the estimated location of each ROI on the CCD to form a basis set of images—one image for each target. Each frame from the time-series stack of images was decomposed into a linear superposition of this basis set using a least-squares fitting, plus one image for background estimation. Formally this was accomplished by creating a matrix of *N* images,

(3) $$B=\left[\begin{array}{ccc} B_{i}\left(1\right) & \ldots & B_{N}\left(1\right)\\ \ldots & \ldots & \ldots \\ B_{i}\left(m*n\right) & \ldots & B_{N}\left(m*n\right) \end{array}\right]$$

where each column is one *m*× *n* image, lexicographically ordered in a column, representative of the expected pattern from the ROI (known from the deterministic illumination). In principle, these images can be given by simulation or found experimentally. Later acquisition of the experimental image, ***I***(*t*), at time *t* (see for example, Figure 1E) can then be characterized by,

(4) I(t)=B·W(t)+n

to describe the image formation where ***n*** is additive random noise. The least-squares fitting,

(5) $$\hat{W}\left(t\right)=\min_{W(t)}{\left\|I\left(t\right)-B\cdot W\left(t\right)\right\|}^{2}$$

quickly yields the individual fluorescence from each target, *w*_*i*_(*t*).

Any systematic movements of the sample would be easily detected as synchronous changes across multiple ROIs, and would not be expected to show the characteristic fast rise and slow decay of calcium transients resulting from action potentials. No such atypical fluorescent changes were detected during our experiments, although controls (gently tapping the microscope stage) showed we can easily detect and distinguish such events.

### Animal experiments

All animal experiments were performed in accordance under approved protocols following the regulations and guidelines of Columbia University's IACUC and ICM. Mice experiments were performed in C57BL/6 mice aged P11 to P60 using 400 μm thick coronal slices loaded with FURA-2AM. Zebrafish experiments were performed at ages P6-P8 and were performed in accordance with the regulations and guidelines of Columbia University and the Howard Hughes Medical Institute. The zebrafish sample is held fixed within a bead of 2% low-melting point agarose to reduce motion artifacts.

## Results

**Figure 3:**
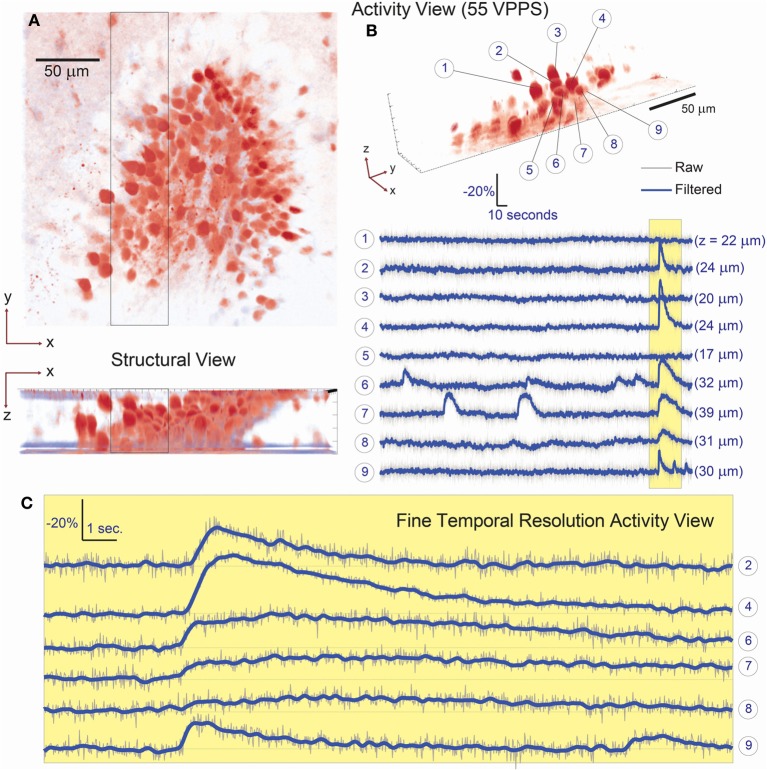
**Simultaneous 3D imaging of hippocampal neuronal activity *in vitro* at 55 volume projections per second. (A)** 3D structural data acquired by two-photon raster-scanning image stack. **(B)** 3D functional imaging with single cell resolution, where 9 of 107 total cells are selected from the 3D volume [boxed region of **(A)**] and their respective activity is given in **(B)**. Despite simultaneous burst activity in 5 of the neighboring cells, independent calcium transients are detectable. The axial location of each cell is given in parenthesis behind the respective fluorescence trace. **(C)** A fine-temporal resolution view of the burst activity highlights the variability in both the temporal and amplitude modulation of the calcium transients.

**Figure 4:**
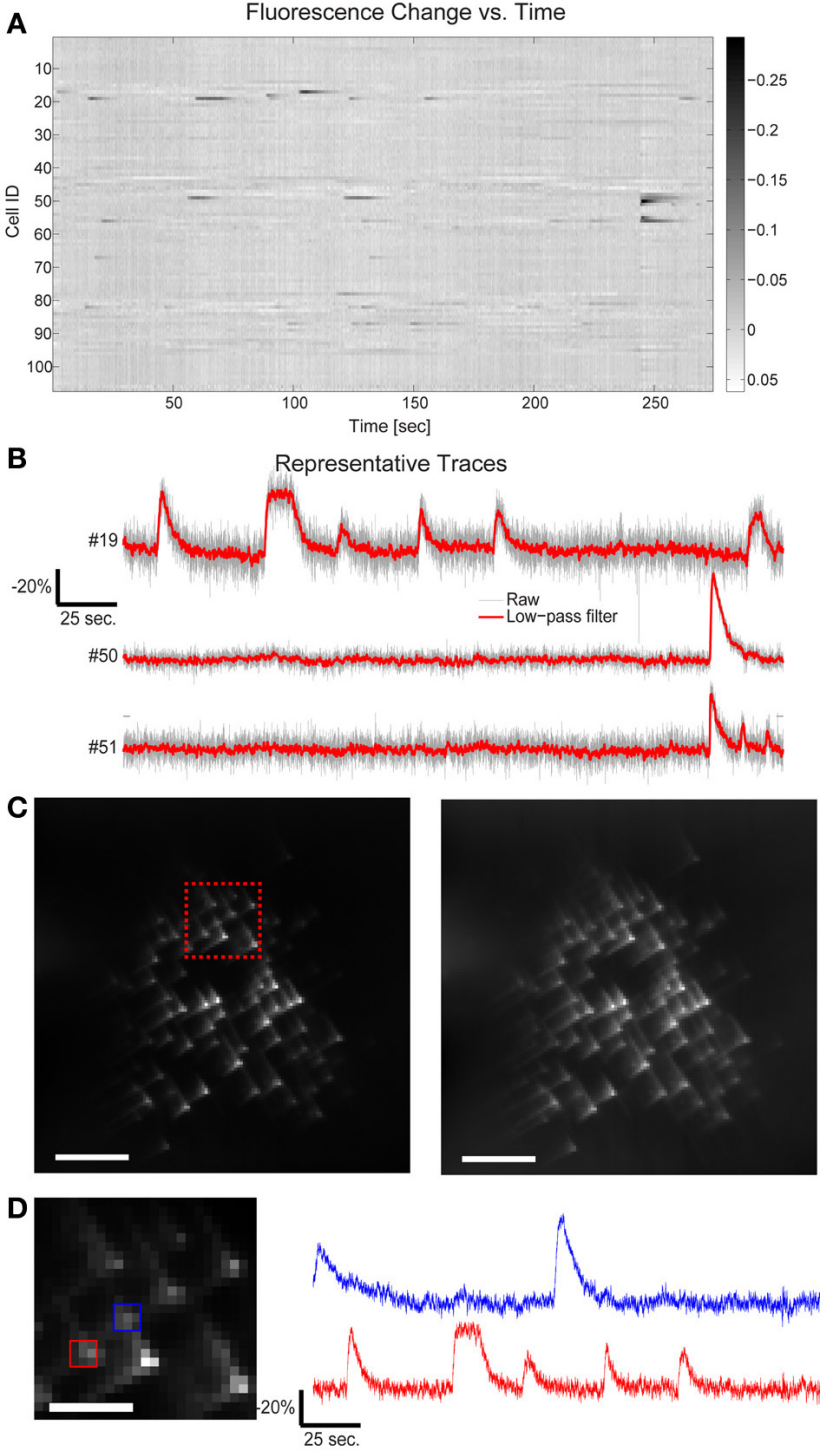
**Fluorescence change vs. time for all targets in Figure 3**. **(A)** 109 cells have been targeted by 109 focal spots in the 3D volume and the relative fluorescence change of each targeted location is displayed. Data has been low-pass filtered by a convolution with a Gaussian filter of σ = 92 ms. **(B)** A few representative traces indicate the SNR available by this method. **(C)** Five second average of the raw acquired images, illustrating both the characteristic cubic-phase aberration and the readily identifiable individual targets. Left image red inset shows area magnified in 4d, right frame is the same image, contrast adjusted to highlight dim features. Scale bar is 40 μm in both images. **(D)** Magnified area of image in **(C)**, showing dense targeting, and again illustrating the cubic-phase aberration. Scale bar is 15 μm. Red and blue boxes indicate ROIs used to generate traces shown on the right, using “traditional” DF/F signal extraction, without fitting. The red trace corresponds to cell #19 in **(B)**, and displays nearly identical absolute modulation and SNR. Note the near absence of crosstalk between the traces, although laterally, the two sources are separated by ~10 μm. Traces are low-pass filtered as in **(A)**.

We first demonstrated the system capabilities using calcium indicators *in vitro*, in acute mouse hippocampal slices to demonstrate single-cell resolution. Sections of the dentate gyrus were selected for 3D imaging, specifically because this brain structure provides a challenging opportunity with its dense ensemble of cells (Figure 3A). Recent work has reported the presence and importance of functional cell-cluster activity with 2D imaging in the hippocampus (Muldoon et al., 2013; Ramirez et al., 2013), however these events have never been observed in 3D. We first present an example of the dense, three-dimensional packing of cells in this structure, which was acquired by conventional sequential raster scanning, and was then used to identify the cells for targeting (Figure 3A). Every visually identified cell in the FOV was targeted for illumination by creating and loading the associated hologram pattern on the SLM. In one representative demonstration, data are acquired for 107 such ROIs (an average of <11 mW incident power per spot) and recorded at 55 volume projection images per second (VPPS) with an exposure time of 16.6 ms per frame (Figures 3B, 4). Individually distinguished fluorescence activity from the dense three-dimensional cluster of cells demonstrates that unique signals from individual neurons can be extracted easily, and with high SNR. Before and after one identified burst, independent activity is observed in each cell body regardless of proximity of the targets in space—demonstrating cellular resolution.

This is perhaps unsurprising after examining the acquired cubic-phase images (Figure 4C). Because SLM-based multisite two-photon excitation preserves the exquisite precision and sensitivity of two photon targeting (Nikolenko et al., 2008), and the cubic phase mask provides reasonable energy compactness (blur is small), and is highly efficient (phase-only modulation), even with dense targeting, simple signal extraction procedures provide high performance. An example of this is shown in Figure 4D, which displays a small area of the total image, and shows the DF/F traces extracted with a small ROI over only the bright central lobe of the cubic-phase PSF (not the basis fitting procedure described earlier) for two targeted cells, whose lateral projections are separated by ~10 μm. These traces show very clean signals, with little to no sign of cross contamination, and show modulation nearly identical to the signal extracted using the least squares fitting procedure (Figure 4B, cell #19, also shown in Figure 4D). As a practical note, in our laboratory, we use this as a rule-of-thumb for the overall quality and performance of the imaging system. If the central lobes of the images of each target are identifiable as distinct puncta, we find that simple methods for signal recovery, such as our linear fitting procedure, and in many cases, traditional ROI selection, will be sufficient to extract high quality signatures from each target.

**Figure 5:**
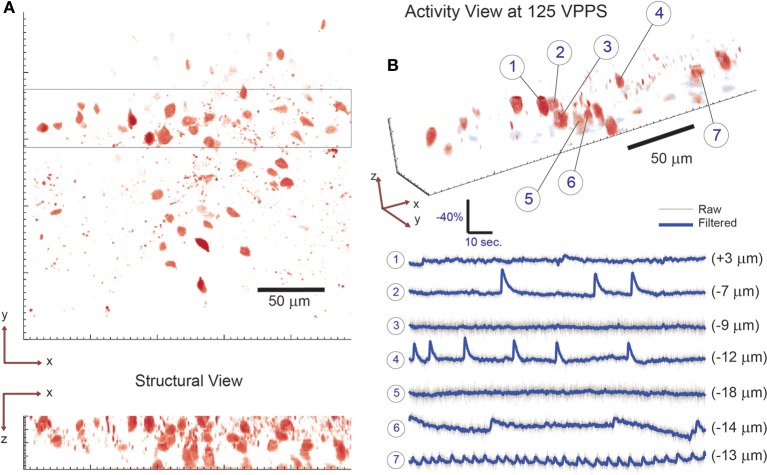
**3D imaging of neuronal activity in dentate gyrus with single-cell resolution at 125 volume projections per second**. **(A)** The location of neuronal cells within the volume of interest is determined by collecting a two-photon raster-scanning image stack and identifying cell bodies. To demonstrate 3D sensing capability with single-cell resolution, 7 neighboring cells are selected from the 3D sub-volume highlighted in **(A)** and their respective activity is shown in **(B)**. Note that neighboring cells are seen to have independent activity—confirming single-cell resolution. The axial location of each cell is given in parenthesis behind the respective fluorescence trace. 58 total targets were monitored (out of 61 total labeled cells identified via visual inspection) throughout the tissue volume.

**Figure 6:**
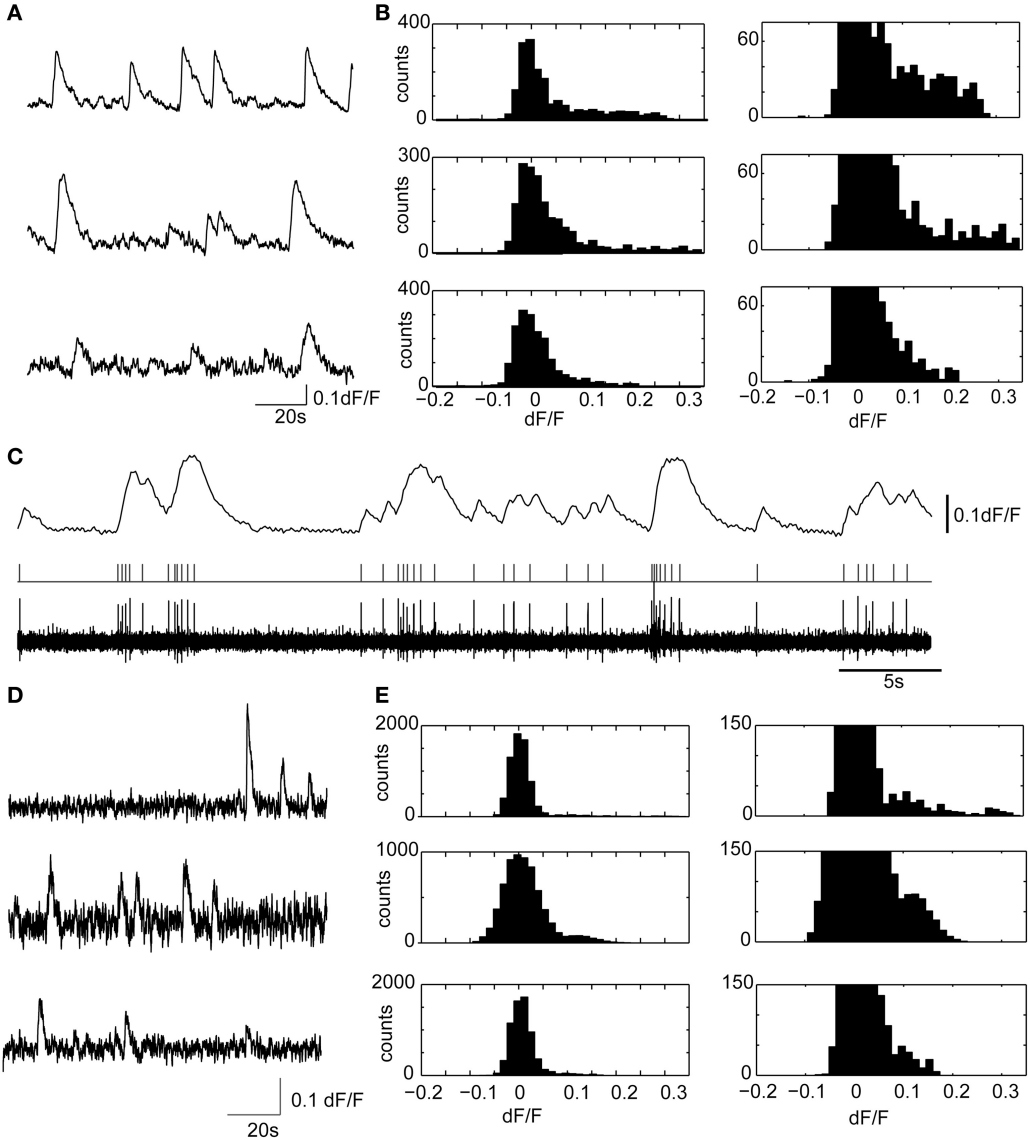
**Calcium transients in hippocampal slices display similar amplitudes and kinetics with confocal microscopy and volume projection 3D imaging**. **(A)** Example calcium transients from three representative granule cell ROIs, acquired from the dentate gyrus, using a spinning disk confocal microscope. The sample preparation was identical to the brain slice experiments described in the main text. Only a short 2 min segment of the data is shown. Data were acquired at 20 fps. **(B)** (Left) dF/F distributions for the three ROIs shown in **(A)**. On the right is the y-axis zoomed histogram, shown to highlight the positively skewed dF/F distributions characteristic of active neuron spiking. **(C)** A representative experiment showing the ability of Fura-2AM calcium transients to faithfully resolve action potential firing in a cell attached recording. The deconvolved calcium dynamics (above) from a cell recording in cell attached are shown (below) with the filtered (0.2–4 kHz) cell attached recording and threshold crossing spikes. Note the ability of the Fura-2AM to resolve single action potential firing, which was usually accompanied by a 5% dF/F. Therefore, in most experiments, single, or at minimum doublet spiking would be detected using calcium imaging. **(D,E)** The same as **(A,B)**, but with three examples of ROIs measured using the 3D volume projection imaging system described in the manuscript. Data displayed were acquired at 55VPPS, and smoothed with a Gaussian filter to match the sample rate of the confocal images. Note the similar shape calcium transients **(D)**, and dF/F distributions **(E)**. The different size calcium dynamics and the dF/F distributions suggest that single or at minimum doublet spiking can be resolved with the volume projection imaging technique.

A key advantage of this method is that minute differences in the onset timing and calcium dynamics of the 3D ensemble can now be resolved with high SNR even at these high temporal sampling rates (Figures 3C, 4). In slices from older animals, with less dense labeling, we demonstrated this method at sampling rates up to 125 VPPS (<20 mW incident power per spot and 6.3 ms exposure times) at single-cell resolution and with high SNR (Figure 5). We have compared the distribution of fluorescent changes and activity patterns collected using the VPI approach to those recorded with a long established imaging method (one photon, laser-illuminated spinning disk confocal, with an EM-CCD, recording at 20 fps) that were accompanied with cell-attached recordings (Figure 6). The recordings also match prior measurements from our laboratory, where ground truth sensitivity to single action potential was clearly demonstrated using the original planar SLM microscope with Fura-2 loaded cortical slices (Nikolenko et al., 2008). Although this is indirect evidence, we believe this indicates that we should be sensitive and have sufficient SNR in most instances to record single action potentials. Co-firing dentate gyrus cells likely represent ensembles of hippocampal neurons involved in pattern separation (Muldoon et al., 2013; Ramirez et al., 2013). Our technique thus allows for the visualization of these activity patterns with unprecedented spatio-temporal precision.

**Figure 7:**
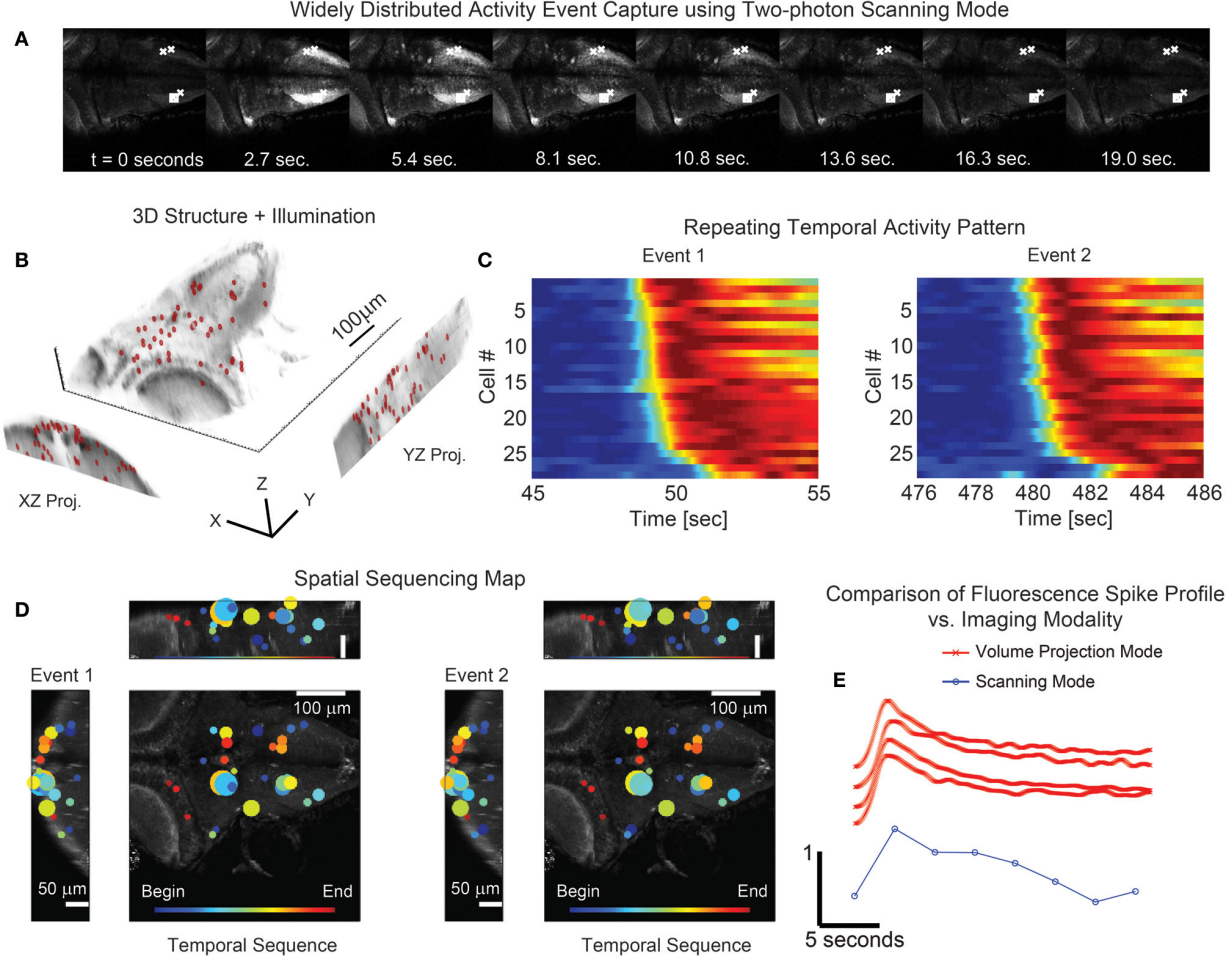
**Simultaneous 3D imaging of neuronal activity of zebrafish *in vivo* at 30 volume projections per second. (A)** Conventional single plane two-photon raster-scan data acquisition reveals sequences of coordinated whole-brain activity. **(B)** 49 targets are distributed throughout an acquisition volume of 284 × 270 × 114 μm to sample activity in 3D. **(C)** Multiple repetitions of these events occur and exhibit similar time-courses. **(D)** The associated spatial patterns of the events confirm that the activity has repetitive structure. The size of the marker is indicative of the amplitude of signal modulation at each location. **(E)** Comparison of the fluorescence spike time profile between the two imaging modalities shows the signal of the volume projection technique to be consistent with the two-photon scanning acquisition. The scanning mode data series was taken from the box marked area in **(A)** while the four volume projection mode series' were taken from the x marked areas. Note that the fluorescence signal has been normalized in each time series of **(C)** and **(E)** for visualization.

We also reconstructed the *in vivo* neuronal activity of the larval zebra fish (P7) with GCaMP5G (Akerboom et al., 2012; Ahrens et al., 2013) to map the temporal record of brain-wide calcium transients at high-speed. Different spatial scales can be accessed with the VPI technique by simply selecting an alternative microscope objective or modulating the strength of the phase aberration present on the wavefront coding element at the pupil (via use of α, in Equation 1). As a reference, we show conventional two-photon galvanometric scanning images that contain brain-wide activity patterns where both individual cells and neuropil are recruited and fluoresce (Figures 7A,E) (see also Ahrens et al., 2013; Panier et al., 2013). In recent work, the temporal resolution of such whole-brain activity mapping was limited by the axial scan speed and the camera frame rate, sampling at 0.8 Hz (Ahrens et al., 2013) or 4 Hz using selective axial planes (Panier et al., 2013). In contrast, the technique proposed here operates simultaneously across axial planes. Moreover, VPI has no moving parts and therefore exhibits no mechanical acquisition speed limitation, nor the complications that can arise from coupling motion of the system into the sample. For illustration, we targeted 49 randomly distributed cells within a 350 × 350 × 150 μm volume (on average <14 mW incident power per spot). Using these ROIs, a custom hologram was created and loaded to the SLM (red targets, Figure 7B). With the simultaneous multisite two-photon excitation, multiple waves of large scale near-synchronous calcium transients are precisely recorded at 30 VPPS (32 ms exposure times per frame). The monitored active cells in these waves were temporally sorted based on their activity in the first wave, and this same ordering was used to display the activity in the subsequent waves. We observed that ordering is strongly preserved in the subsequent waves, with sub-second precision, despite occurring many minutes later (Figure 7C). It is important to note that this is not an epileptiform or simple activity pattern—the spatiotemporal profile of the activity is dispersed throughout the sample with the cells closest geographically not necessarily having the smallest relative offsets in the onsets of activity (Figure 7D). This is, to our knowledge, the first demonstration of simultaneous three dimensional calcium activity imaging in *in vivo* zebra fish preparations with single cell precision at a temporal resolution sufficient to resolve the dynamics of neural activity patterns.

## Discussion

**Figure 8:**
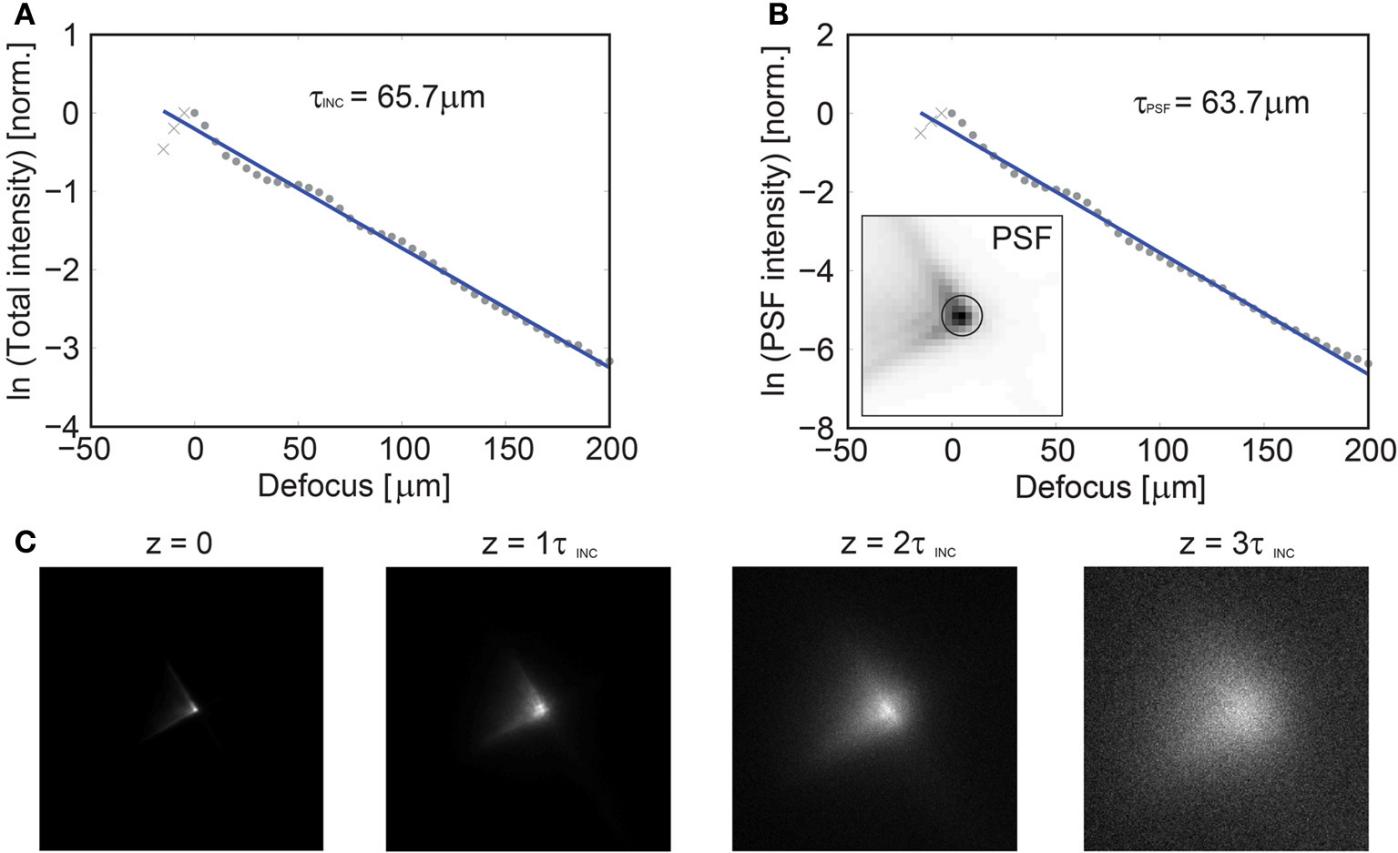
**Axial dependence of the imaging PSF vs. depth in scattering tissue**. Characterization of the imaging performance of volume projection imaging using GCaMP6s labeled tissue at P31 in white matter tissue. GCaMP6s was chosen to provide near uniform labeling throughout the tissue. **(A)** A focused spot was translated axially via a mechanical stage and the resulting collected intensity is reported, yielding a mean scattering length of the incident illumination (λ = 920 nm) of τ_INC_ = 65.7 μm. In an attempt to quantify the loss of the PSF fidelity with increasing scattering, a circular region was defined [centered on the peak intensity, insert **(B)**] and the relative loss of energy was plotted as a function of depth, yielding τ_PSF_ = 63.7 μm **(B)**. An image of the PSF is given as a function of τ_INC_ in **(C)** and the PSF fidelity is observed to degenerate by ~3τ_INC_, at which point the system performance approaches that of a conventional SLM microscope.

Using SLM structured illumination with VPI, we can image the activity of neuronal populations throughout the brain of the larval zebrafish simultaneously. But, although the larval zebra fish is transparent, most living organisms are not. Our method as implemented here still relies on direct optical imaging and is adversely affected in highly scattering environments. This has been a challenge for all optical imaging methods in neuroscience, and the described method is not immune to it. We evaluated the effect of scattering on the PSF used here and believe that this method can be applied up to 2–3 scattering lengths deep providing that a relatively sparse selection of 3D points is used (Figure 8). However, there are active developments which will extend the absolute depth where this method can be successfully applied. First, ongoing improvements in red shifted indicators and light sources, whose longer wavelengths penetrate more deeply through tissue, could allow for significant and sizeable volumetric imaging with high spatial and temporal resolution. With the development of faster SLMs, or external modulation schemes, temporal coding and multiplexing can be added to augment the selectivity at large imaging depths by imposing an a priori temporal structure on each target, allowing for phase locked detection (Ducros et al., 2013). This could be a critical improvement, as it would allow for imaging neurons whose projections fall within the same pixels of the camera (such as neurons directly on top of one another). Additionally, advances in algorithms for signal extraction that jointly consider the recorded spatial and temporal signatures of each source will improve separation for overlapping and delocalized signals (Pnevmatikakis et al., 2013).

Both the illumination and data acquisition are simultaneous and can target multiple ROIs throughout the volume of tissue allowing for parallel activation and imaging. We have demonstrated high SNR recording of calcium transients at speeds of up to 125 Hz, limited by the camera transfer rate. This frame rate and sensitivity is already well matched to the current generation of GFP based genetically encoded voltage indicators, such as Arclight (Jin et al., 2012). Advances in the technologies utilized here, such as higher power lasers and increased pixel count SLMs, along with faster cameras will soon accommodate direct imaging of voltage activity in 3D.

In contrast to high speed acousto-optic deflector microscopy techniques, 3D SLM microscopy has the advantage of decoupling the pixel dwell time (time allotted to collect fluorescence signal) from the number of targets. This is an important distinction, and deserves attention. Admittedly, the best fast AOD systems are currently capable of high performance, and set a mark by which all alternative high speed approaches are measured. However fast the performance, we believe they will be difficult to scale significantly beyond their current levels because with very high speed random access approaches, the total collected fluorescence signal per target is limited by the relatively low duty cycle per location and the maximum emission rate of the fluorophore (a physical characteristic of the particular chromophore). Though each target could theoretically receive the full output of the laser, simply increasing the intensity of illumination yields diminishing returns because of fluorophore saturation and continued high intensity excitation will lead to increased photodamage and bleaching, rather than increased signal, along with a loss of spatial resolution (Hopt and Neher, 2001). With SLM microscopy, the issue is different. Each target has very high duty cycle with respect to the sampling rate (true simultaneous multisite illumination), but with lower average instantaneous power—multiplexing the beam results in diminished fluence per target, with a corresponding reduction in total fluorescence per target. In this case, what limits the total number of targets is the overall laser power available, which can be arbitrarily increased until the overall power deposition over the entire FOV exceeds the total acceptable heat load for the sample. We note that with programmable SLMs, one can achieve the optimal synthesis of both methods with simultaneous, multisite random access targeting, offering tremendous flexibility in monitoring activity. Also, while we describe two photon SLM-based multiple beamlet excitation here, any predetermined structured illumination can be used.

In conclusion, we present a technique for fast, simultaneous, two-photon optical data acquisition of neuron activity which is distributed throughout three dimensions. This has been demonstrated in different animal preparations, both *in vitro* and *in vivo*, that are relevant for neuroscience. The combination of structured illumination with volume projection imaging appears to us a promising platform for future work on brain activity mapping (Alivisatos et al., 2012).

### Conflict of interest statement

The authors declare that the research was conducted in the absence of any commercial or financial relationships that could be construed as a potential conflict of interest.
